# New Appliances and Things Medical

**Published:** 1906-02-10

**Authors:** 


### NEW APPLIANCES AND THINGS MEDICAL.

### THE SPARKLOGENE.

(Aerators, Limited, Angel Road, Edmonton, London, N.)

We have received from Aerators, Limited, one of their new three-pint Sparklet seltzogenes. In shape the apparatus somewhat resembles the old-fashioned seltzogene, but is simpler in construction. In general, the sparklogene supplies a simple and absolutely clean method of aerating water, wine, or other beverage, without any such tedious and troublesome process as was required with the old form of seltzogene. All that is necessary is to place the liquid which it is desired to aerate into the apparatus, and to charge it with three of the well-known Sparklet bulbs. Not only is this a simple matter, but it is a very brief one; moreover, the aerated beverage is ready for immediate consumption, whereas with older forms of domestic aerators it took a considerable time to complete the process. People nowadays are coming more and more to realise that the only way to render water an absolutely safe beverage is to boil it. And the only way to get rid of the "flatness" of boiled water and to make it palatable is to aerate it. Under these circumstances the manifest advantages of the Sparklogene are sure to commend it strongly to the intelligent householder. The price of the apparatus complete is 7s. 9d., and the perishable portions can be separately renewed.

